# Perioperative Prophylaxis in Pediatric Factor VII Deficiency: Experience From Rabat Children’s Hospital

**DOI:** 10.7759/cureus.107814

**Published:** 2026-04-27

**Authors:** Chaymaa El Messari, Zineb Isfaoun, Naoual El Ansari, Meryem Lakhrissi, Maria Kababri, Amina Kili, Laila Hessissen, Mohamed Elkhorassani

**Affiliations:** 1 Hemophilia and Inherited Bleeding Disorders Treatment Center, Pediatric Hematology and Oncology Center, Ibn Sina University Hospital Center, Mohammed V University, Rabat, MAR

**Keywords:** bleeding disorders, factor vii deficiency, hemostasis, pediatric surgery, perioperative prophylaxis, recombinant activated factor vii

## Abstract

Background: Factor VII (FVII) deficiency is a rare inherited bleeding disorder characterized by heterogeneous clinical manifestations and unpredictable bleeding risk. Surgical procedures in affected children may expose them to significant perioperative bleeding, highlighting the importance of appropriate prophylactic management.

Objective: The objective of this study is to evaluate postoperative hemostatic outcomes and hemorrhagic complications according to the type of surgery and perioperative prophylaxis in children with FVII deficiency.

Methods: We conducted a retrospective review of the medical records of patients with confirmed FVII deficiency who underwent surgical procedures. Collected data included demographic characteristics, baseline FVII activity levels, type of surgery, perioperative prophylaxis, and postoperative outcomes.

Results: Eight children aged 4-6 years were included. Baseline FVII activity levels ranged from 1.6% to 22%. Surgical procedures included circumcision, hernia repair, tonsillectomy, and dental extractions. Prophylaxis consisted mainly of recombinant activated factor VII at 30 µg/kg administered 30 minutes before surgery, while one patient received fresh frozen plasma (FFP) at 20 mL/kg. Postoperative bleeding was minimal overall. Two patients developed complications: a scrotal hematoma in the patient who received FFP prophylaxis and a bleeding episode revealing previously undiagnosed FVII deficiency. No thrombotic events were observed.

Conclusions: Perioperative prophylaxis with rFVIIa was associated with favorable hemostatic outcomes in children with congenital FVII deficiency undergoing surgery. A single preoperative dose appeared sufficient for minor procedures in this cohort; however, these findings should be interpreted with caution, given the small sample size (n=8), and require confirmation in larger studies.

## Introduction

Congenital factor VII (FVII) deficiency is the most common among rare inherited coagulation disorders. However, its clinical expression is highly variable, ranging from mild mucocutaneous bleeding to severe, potentially life-threatening hemorrhages [1,2]. Inherited FVII deficiency has an estimated incidence of 1/300,000-1/500,000 in the general population and follows an autosomal recessive pattern of inheritance [3,4]. The prevalence of severe forms may be higher in countries where consanguineous marriages are frequent [5].

Plasma FVII activity levels do not reliably correlate with bleeding severity, making perioperative management particularly challenging and requiring individualized risk assessment [6]. In pediatric practice, commonly performed procedures such as circumcision, dental extractions, and hernia repair may expose affected children to substantial bleeding risk. Careful preoperative evaluation, appropriate replacement therapy, and close postoperative monitoring are therefore essential [7].

Despite available case reports and registry data, evidence regarding perioperative prophylactic strategies and surgical outcomes in children with congenital FVII deficiency remains limited, particularly in real-world pediatric settings from low- and middle-resource contexts.

This study reports a series of eight pediatric patients with congenital FVII deficiency who underwent surgical procedures.

The primary objective of this study was to evaluate the efficacy and safety of perioperative replacement therapy, including hemostatic outcomes and postoperative bleeding complications. The secondary objective was to describe the clinical and biological characteristics of children with FVII deficiency managed at our center.

## Materials and methods

Study design

This retrospective study was conducted at the Hemophilia and Inherited Bleeding Disorders Treatment Center (CTHMH) at Rabat Children’s Hospital, within the Department of Pediatric Hematology and Oncology, affiliated with Ibn Sina University Hospital Center and Mohammed V University, Rabat, Morocco.

Study population and sample size

The study included pediatric patients with a confirmed diagnosis of congenital FVII deficiency followed at our center between 2011 and 2025. During this period, a total of 31 pediatric patients with confirmed congenital FVII deficiency who were diagnosed and/or followed at our center were identified. These patients do not represent all hospital admissions, and not all admitted patients underwent systematic evaluation for hemophilia or inherited bleeding disorders. Among the 31 confirmed FVII-deficient patients, only eight underwent surgical procedures and were therefore included in the perioperative analysis.

Given the rarity of the condition, all eligible surgical cases during the study period were included. The small surgical sample size (n=8) is acknowledged as a limitation.

Case definition

Patients were considered eligible if they had confirmed FVII deficiency, defined by a baseline plasma FVII activity level (FVIIc) below the normal reference range, regardless of prior bleeding history. Both male and female patients were included.

Medical records were excluded if key variables required for analysis were missing, including baseline FVII activity level, prophylactic treatment details, or postoperative outcome data.

Data collection

Data were retrospectively extracted from medical records and included demographic characteristics (age and sex), circumstances and age at diagnosis, laboratory parameters (prothrombin time, activated partial thromboplastin time, and baseline FVII activity level), and the type of surgical procedure performed.

Information on personal bleeding history prior to surgery, perioperative prophylaxis (including type of replacement therapy, dosage, and timing of administration), postoperative bleeding events, procedure-related complications, and associated comorbidities was also collected.

The single patient who received fresh frozen plasma (FFP) underwent surgery in 2012, when recombinant activated factor VII (rFVIIa) was not yet available at our center; all subsequent patients received rFVIIa-based prophylaxis according to institutional practice.

Definitions and study limitations

Perioperative bleeding was defined as any clinically evident bleeding occurring during or after surgery requiring additional hemostatic intervention, prolonged observation, or associated with a documented postoperative bleeding complication.

Effective prophylaxis was defined as the absence of significant perioperative bleeding and the absence of need for unplanned additional replacement therapy. Statistical analysis was descriptive, given the small surgical sample size. Given the retrospective design, potential selection bias and information bias were considered and are acknowledged as study limitations.

## Results

A total of 31 pediatric patients with confirmed congenital FVII deficiency were identified during the study period. Among them, only eight patients underwent surgical procedures and were therefore included in the perioperative analysis. The remaining patients did not undergo any surgical intervention during the study period.

The perioperative characteristics and outcomes of these patients are summarized in Table 1.

**Table 1:** Case Series of Surgical Procedures in Pediatric Factor VII Deficiency FFP: Fresh frozen plasma; rFVIIa: Recombinant activated factor VII;  FVIIc: Baseline plasma FVII activity level; FVII: Factor VII

Case	Circumstances of discovery	Age of diagnosis	Sex	PT	aPTT	FVIIc(%)	Type of surgery	Bleeding history	Pre-op prophylaxis	Product (dose)	Bleeding outcome	Remarks
1	Preoperative workup	2 years	M	46%	1	21.1	Hernia repair 2012	None	Yes	FFP 20ml/ kg	scrotal hematoma	FFP four days post surgery
2	Bleeding after the loss of a deciduous tooth	6 years	F	14%	1.16	2.1	Dental extraction	Mild mucosal bleeds	Yes	50 µg/ kg rFVIIa	Minimal bleeding, considered insignificant	Dose given 30 minutes preop
3	Bleeding after circumcision	6 years	M	14%	1.6	17	Circumcision	None	None	None	Very short	Diagnosed after surgery
4	Preoperative workup	3 years	M	44%	1.06	22.09	Tonsillectomy	None	Yes	50 µg/ kg rFVIIa	None	Associated with sickle cell disease
5	Preoperative workup	5 years	M	23.1%	1.04	1.6	Hernia repair	None	Yes	50 µg/ kg rFVIIa	None	Dose given 30 minutes preop
6	Preoperative workup	2 years	M	46%	1.08	16	Circumcision	None	Yes	50 µg/ kg rFVIIa	None	Dose given 30 minutes preop
7	Preoperative workup	3 years	M	59%	1.15	28	Circumcision	Mild mucosal bleeds	Yes	35 µg/ kg rFVIIa	None	Dose given 30 minutes preop
8	Preoperative workup	2 years	M	15%	1.26	1.9	Circumcision	None	Yes	35 µg/ kg rFVIIa	None	Dose given 30 minutes preop

Most operated patients were male (seven boys and one girl), with ages ranging from 4 to 6 years at the time of surgery.

In these eight patients, the diagnosis of FVII deficiency was established either during preoperative assessment or following bleeding events, such as after circumcision, dental extraction, or exfoliation of deciduous teeth.

Baseline FVII activity levels ranged from 1.6% to 22%. According to factor activity levels, five patients had mild deficiency (15-30%), while three had severe deficiency, with levels between 1% and 2%. Prothrombin time values ranged from 14% to 59%.

The surgical procedures performed included four circumcisions, two hernia repairs, one tonsillectomy, and one dental extraction.

A history of mild mucosal bleeding was reported in two patients prior to surgery and did not correspond to postoperative bleeding events.

Perioperative prophylaxis was administered in most cases, predominantly using rFVIIa at doses ranging from 30 to 50 µg/kg. One patient received FFP at a dose of 20 mL/kg in 2012 due to the unavailability of rFVIIa at that time. Prophylaxis was generally administered approximately 30 minutes before surgery.

No postoperative bleeding events were observed in patients who received rFVIIa prophylaxis. However, two patients experienced hemorrhagic complications: one developed a scrotal hematoma following hernia repair after receiving FFP prophylaxis, and another presented with bleeding after circumcision, which led to the diagnosis of previously unrecognized FVII deficiency.

## Discussion

Plasma FVII levels do not reliably correlate with the severity of the bleeding phenotype, making perioperative risk assessment challenging and supporting a prophylactic approach guided primarily by clinical phenotype rather than factor levels alone [4,5]. In our cohort, the predominance of male patients reflects the frequent clinical presentation at the time of circumcision, although both sexes are equally affected by the disorder [6,7].

Diagnosis was commonly established during preoperative screening or after mild bleeding episodes, such as dental extractions or exfoliation of primary teeth, highlighting the marked phenotypic variability of congenital FVII deficiency. In three patients with FVII levels below 2%, the diagnosis was made at around five years of age during routine preoperative evaluation.

rFVIIa is currently regarded as the treatment of choice for congenital FVII deficiency because of its targeted hemostatic action [8]. In our cohort, perioperative prophylaxis based mainly on rFVIIa was associated with the absence of significant postoperative bleeding events. However, given the small sample size and retrospective design, these findings should be interpreted with caution.

In accordance with current recommendations, prophylaxis was administered approximately 30 minutes prior to surgery [9,10]. In our series, a single perioperative dose of rFVIIa appeared sufficient to achieve adequate hemostasis in minor surgical procedures, without the need for additional postoperative injections. This approach required close postoperative monitoring and may help reduce the number of administered doses and overall treatment cost.

These findings are in line with data from international registries reporting effective hemostatic control with rFVIIa in most surgical settings. However, due to the short half-life of rFVIIa (approximately 2-3 hours), repeated dosing may be necessary in major surgical procedures [9,10].

Although rFVIIa is generally well tolerated, the potential risk of thrombotic events should be considered, particularly in patients with associated comorbidities or in older populations [10]. In our pediatric cohort, no thrombotic complications were observed, although the limited sample size precludes definitive conclusions regarding safety.

Our findings suggest that surgical procedures may be performed with favorable outcomes in children with FVII deficiency when appropriate prophylactic measures are implemented. Preoperative evaluation should include a detailed bleeding history, baseline FVII activity, and the type of planned surgery. Individualized management and close coordination between hematology, surgical, and anesthesia teams remain essential. Adjunctive therapies, such as tranexamic acid and local hemostatic measures, may be considered, particularly in minor or mucosal procedures [9].

Despite the clinical relevance of these observations, this study has several limitations, including its retrospective design, small sample size, single-center setting, and heterogeneity of surgical procedures, which may limit the generalizability of the findings [11].

## Conclusions

Children with congenital FVII deficiency may undergo surgical procedures with favorable outcomes when an individualized perioperative prophylactic strategy is implemented. In our cohort, preoperative administration of rFVIIa was associated with effective hemostatic control and a low rate of bleeding complications. For minor surgical procedures, a single preoperative dose of rFVIIa appeared sufficient in our experience; however, this observation should be interpreted cautiously, given the limited sample size. These findings should be considered preliminary and require confirmation in larger prospective studies. Rigorous preoperative assessment, close peri- and postoperative monitoring, and multidisciplinary collaboration between hematology, surgery, and anesthesia teams remain essential to optimize surgical outcomes in children with congenital FVII deficiency.
